# Practical Strategies for Discovering Regulatory DNA Sequence Motifs

**DOI:** 10.1371/journal.pcbi.0020036

**Published:** 2006-04-28

**Authors:** Kenzie D MacIsaac, Ernest Fraenkel

Many functionally important regions of the genome can be recognized by searching for sequence patterns, or “motifs.” Aside from the genes themselves, examples include CpG islands, often present in promoter regions, and splice sites that denote intron/exon boundaries. Other motifs of great interest correspond to sites bound by regulatory proteins. Differential expression of genes in response to environmental and developmental cues depends on the action of these proteins, which are also known as transcription factors. Identifying the regulatory motifs bound by transcription factors can provide crucial insight into the mechanisms of transcriptional regulation. However, the search for these sites is challenging because a single regulatory protein will often recognize a variety of similar sequences. In this tutorial, we review computational techniques, termed “motif discovery,” to learn representations of regulatory motifs from sequence data. In Figure 1, we present an overview of the basic workflow in a motif discovery analysis and some practical strategies for successfully mining sequence data for biologically important regulatory motifs. In the remainder of this tutorial, we discuss the main challenges associated with motif discovery in detail, and we review recent developments for addressing these challenges.

**Figure 1:**
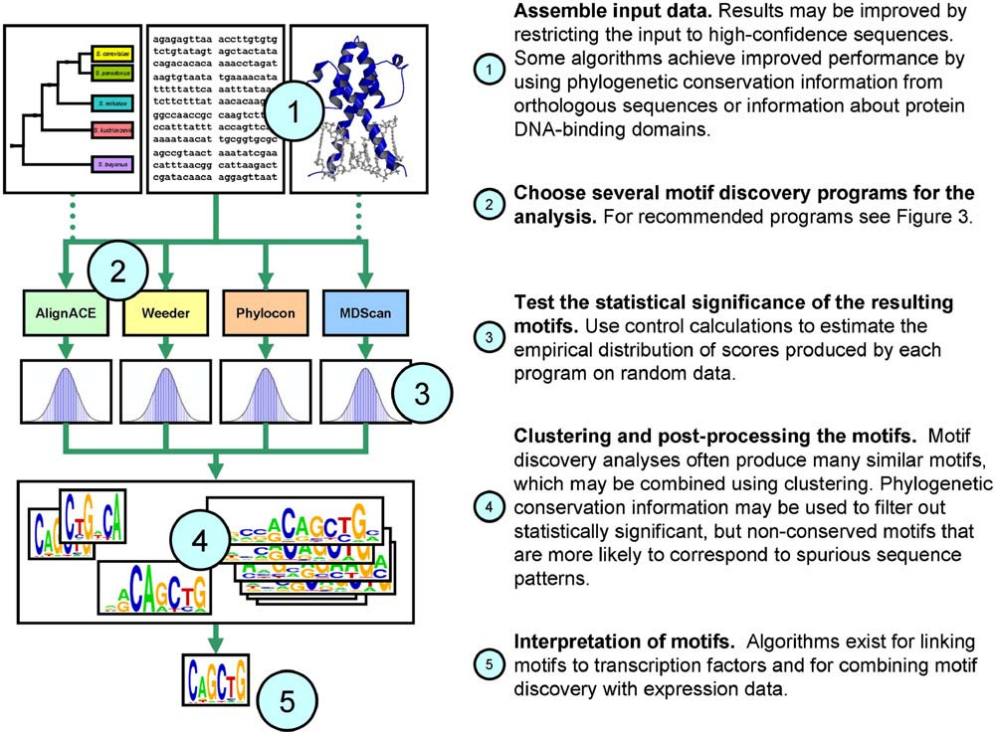
Motif Discovery Workflow

## Theoretical Considerations

### 

#### Motif models.

There are many ways of representing the sequence specificity of a protein, and the choice of a particular representation is often determined by considerations such as simplicity, interpretability, representational power, or computational convenience. Perhaps the simplest way of representing a motif is by using a consensus sequence of preferred nucleotides (adenine [A], cytosine [C], guanine [G], or thymine [T]). A motif is then simply a short word embedded in a longer DNA sequence. Degeneracy in the binding specificity of a protein can be incorporated using the ambiguity codes (purine [R], pyrimidine [Y], strong [S], weak [W], keto [K], amino [M], and any nucleotide [N]) [1]. A number of methods for generating consensus sequences from data are possible, and several methods have been compared by Day and McMorris [2].

Another widely used motif model is the position weight matrix (PWM). In this formulation, the motif is represented as a matrix of nucleotide scores indexed by letter and position [3]. In a PWM, the nucleotide observed at a particular position in the motif is assumed to be independent of the nucleotides observed at other positions [4]. A closely related approach models a motif as a matrix of nucleotide probabilities, where each position is represented using a multinomial distribution over observed nucleotides. Motifs represented in this manner can be visualized conveniently using sequence logos. A sequence logo consists of an ordered stack of letters, where a letter's height indicates the information it contains at that position [5]. For example, a nucleotide that appears 100% of the time at a particular position reduces our uncertainty about the binding site sequence by two bits, and therefore will have a height of two bits in the sequence logo. The nucleotide frequencies observed at different positions in a set of binding sites can be related to the theoretical contribution of a particular nucleotide to the free energy of protein binding [4,6,7].

Consensus sequences and simple matrix models ignore some of the complexity of protein–DNA interaction. Dependencies between nucleotides at different positions in protein binding sites have been observed [8,9]. Several motif models have been proposed that take into account the possibility of positional correlations. Zhou and Liu modeled a motif using a generalized weight matrix that could incorporate pairwise dependencies [10]. Barash and colleagues used Bayesian networks to model motifs, allowing for the incorporation of arbitrary dependencies between positions [11]. Several other representationally powerful models have been proposed that can incorporate dependencies, including boosted classifiers [12] and a hidden Markov Dirichlet multinomial model [13].

While it is possible to use arbitrarily complex motif models to represent a transcription factor's binding specificity, increasing the model complexity requires more data to estimate the model's parameters. If data are limited, as they often are, complex models may overfit the data and subsequently yield a poor representation of the factor's true specificity. An important study by Benos, Bulyk, and Stormo suggested that while the consensus sequence and PWM may not fully capture all the subtleties of a protein's binding specificity, these simple and easily interpretable models usually provide a very good approximation to reality [14].

#### Algorithms for motif discovery.

The motif discovery problem can be formulated in several ways, but the most common formulation is as follows: we have a set of DNA sequences that are believed, a priori, to be co-regulated and thus likely to be bound by one or more regulatory proteins. We wish to learn the parameters of motifs that could explain this binding. To a large extent, the algorithm used to perform motif discovery dictates which type of motif model will be used. The algorithmic approaches that have been used to tackle this problem may be grouped broadly into two categories: enumerative methods and alignment-based methods.

Enumerative methods typically involve exhaustive enumeration of words up to some maximum size in a dataset, and are thus best suited to consensus sequence motif models. Once the words are cataloged, they can be scored using an appropriate measure of statistical significance, and the most statistically significant motifs are then reported. The computational time complexity of enumerative methods is approximately *O(NmA^e^L^e^),* where *N* is the number of sequences, *m* is their length, *A* is the alphabet size, *L* is the motif length, and *e* is the number of errors allowed in a match to a catalog entry [15]. Many enumerative methods use trade-offs on the alphabet size and the number of allowable errors to make these searches computationally feasible [15–18]. Recently, dictionary-based motif discovery methods have been proposed that are related to word enumeration methods, but which incorporate a probabilistic model of how sequences are generated from a dictionary of possible words [19–23].

Alignment methods take on a wide variety of forms, but often involve development of a probabilistic model of the observed sequence data and optimization to find motifs common to all input sequences. The MEME program, for example, treats a particular sequence as arising from a mixture model in which the small window of sequence containing the motif is generated from a motif model—represented by a probability matrix—and the rest of the sequence is treated as arising from a Markovian background [24]. The generative model describes a family of parameterized probability distributions, and the motif is simply a parameter of this distribution. Any number of optimization techniques may be used to search for the parameter setting that maximizes the likelihood of the observed sequence data. Two frequently used techniques to perform this search are the expectation-maximization (EM) algorithm and Gibbs sampling.

The EM algorithm is a general approach for maximizing a likelihood function with hidden variables [25]. In the case of alignment-based motif discovery applications, the hidden variables are the locations of the motif in the set of input sequences. EM consists of two steps: in the E-step, the expected likelihood of the observed sequence data is calculated based on the current setting of the parameters, and in the M-step, the parameters are updated to maximize the expected-likelihood function. EM is a local optimization procedure that is guaranteed to monotonically improve the expected likelihood, but it is sensitive to its initialization point and is therefore not guaranteed to converge to the global maximum. For this reason, motif discovery programs that use EM will typically restart the optimization from many distinct initialization points to improve the chances of converging to the global maximum. Multiple restarts also improve the chances of finding biologically relevant motifs that may not necessarily correspond to the global maximum. Interesting heuristics for selecting reasonable initialization points have been developed [26,27].

Gibbs sampling is a general technique for performing probabilistic inference [28]. Like EM, it is well suited to problems such as motif discovery with incomplete information. However, unlike EM, it is an undirected and global search over a parameterized distribution. In the context of motif discovery, Gibbs sampling involves drawing random samples of the hidden variables (typically motif location) from a distribution. The parameters are reestimated based on the randomly generated samples, and then sampling is repeated. The global nature of the Gibbs sampling search comes at significant computational cost, and the algorithm may have to be run for many iterations to obtain adequate representations of the complicated likelihood surfaces typically encountered in motif discovery.

## Motif Discovery in Practice

### 

#### Co-regulated genes can be identified in a number of ways.

Motif discovery typically begins with a group of putatively co-regulated genes. These co-regulated sets are often obtained by using clustering to identify genes that share a functional category or are co-expressed under a number of different experimental conditions. Motif discovery is then performed on the relevant promoter regions [29–34]. Other approaches have been developed that do not necessarily require clustering [35–37]. Chromatin immunoprecipitation (ChIP) data are a second important source of co-regulated genes [38–46]. ChIP-chip experiments measure at low resolution, and, potentially, on a genome-wide scale, the binding of a particular protein to DNA using microarray technology. Analyzing these data with motif discovery programs can reveal motifs representing the specificity of the proteins, and can be used to improve the resolution of the data. Sequences in the bound regions that match the motif are the most likely binding sites. Regions that are bound but do not contain matches to the motif may be sites of indirect regulation or may be spurious binding events, arising from noise in the data. A third type of analysis has focused on genome-wide motif discovery, where potentially important regulatory motifs have been cataloged by examining entire genomes [47–50]. These analyses generally include information about phylogenetic conservation to help identify sequence signals that are conserved at a higher-than-expected rate, and are therefore more likely to be functional.

#### Some factors affecting motif discovery performance.

To understand the factors that affect motif discovery, it is helpful to think of a motif as a signal buried in genomic noise (i.e., the background sequence) [6,51–54]. A motif with very low information content is difficult to distinguish from the background sequence, and therefore has low signal strength. Basic statistical considerations relating to motif frequency and overrepresentation in the dataset also affect performance. Adding false-positive inputs or increasing the length of the input sequences is akin to increasing the amount of noise within which the motif signal is hidden. Another important consideration is the number of input sequences. Hu and colleagues found that, for five separate programs, motif discovery performance leveled off after a certain number of input sequences [52]. In light of these considerations, it appears that a smaller number of high-confidence sequences is preferable to a large number of low-confidence inputs for most motif discovery analyses. It is also advisable to keep input sequences short in order to minimize the amount of uninformative background DNA from which the motif must be distinguished.

Recent advances in experimental technology—such as the newly developed DNA immunoprecipitation with microarray detection (DIP-chip) technique for determining binding specificity [55], densely tiled short oligonucleotide arrays used in ChIP-chip [56], and computational techniques for increasing the resolution of these data [57]—may ultimately prove to be very valuable in providing higher-quality input for motif discovery.

#### Using multiple motif discovery programs improves performance.

The emerging consensus from a number of comparative studies is that no single program is superior for all datasets. Harbison and colleagues used a suite of six different motif discovery tools to analyze a collection of ChIP experiments for 172 transcription factors in Saccharomyces cerevisiae. They found that no program demonstrated clear superiority, and that all programs discovered at least one motif that none of the other programs could recover [39]. In another recent and well-designed study, Tompa et al. assessed the performance of 14 motif discovery programs on a wide variety of real and synthetic datasets [58]. They avoided a common pitfall of many of these types of comparisons by ensuring that the analyses were performed by those with expertise in operating the software. One notable result was that all programs performed well on yeast data; however, their performance degraded significantly when applied to the more complex sequence data in flies and humans. Again, no single program was superior across all performance measures and datasets, although the program Weeder [15] stood out as having significantly better performance than most. A third study by Hu et al. [52] compared the performance of five popular motif discovery programs and again observed comparable performance among all programs. In a formalization of the approach of Harbison et al., Hu and colleagues demonstrated that a significant improvement in performance could be achieved by combining the output of the five programs into a single “consensus ensemble algorithm.” These results underscore the utility of analyzing sequence datasets with several motif discovery tools. The potential benefit of using several programs often more than makes up for the effort associated with combining and postprocessing the results.

#### Recommended methods for scoring motifs.

**Figure 2:**
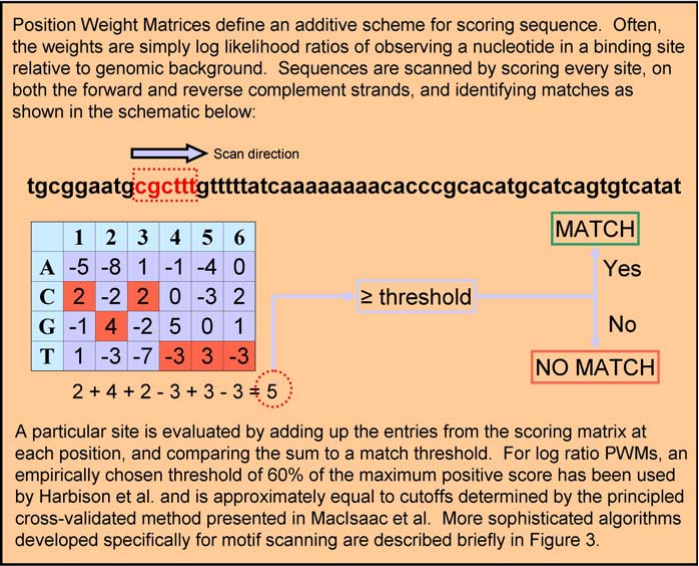
Scanning for Motifs with PWMs

During postprocessing of the motif discovery output, it is valuable to use a consistent scoring metric that allows motifs to be compared and ranked regardless of their source. Any scoring metric relies first and foremost on the ability to scan a sequence to determine whether the motif is present. For motifs represented as a consensus sequence, scanning is accomplished by searching for subsequences that match the consensus word, with a prespecified threshold on the number of allowable errors. For motifs represented with PWMs, it is necessary to specify a method for scoring sites, and also to specify a threshold score that defines a match. Statistically principled methods of assessing cutoff thresholds for motif matches have been presented [59,60]. Scanning sequences for motifs using PWMs is an important problem in its own right [54,59–62], and we present an overview of the basic procedure in Figure 2.

Once a criterion for specifying a match to a motif has been determined, it is possible to evaluate particular motifs learned from a dataset. Various scoring criteria for motifs have been developed, and most motif discovery programs have their own preferred metric for scoring. Most scores involve a measure of information content [63] or statistical overrepresentation [32,64,65]. In our experience, two particularly intuitive and useful scores are the hypergeometric enrichment and the area under the receiver operating characteristic curve (ROC-AUC).

The hypergeometric enrichment score can be used to measure the statistical overrepresentation of a motif [64]. We assume that there are many sequences representing the genomic background from which the input sequences were selected. If for example, motif discovery was performed on a set of Drosophila melanogaster promoters, a suitable background might be the set of all known promoter regions in *Drosophila*. The enrichment score is calculated by counting the number of occurrences of the motif in the input and in the entire background. The hypergeometric *p*-value is the probability that we would observe an equal or greater number of motif occurrences if the input dataset had been drawn randomly and without replacement from the background. The enrichment score is the negative log of this *p*-value [39]. If the motif is highly overrepresented in the input dataset, then the probability of observing a count that large at random will be very small, and the enrichment score will be large.

A receiver operating characteristic (ROC) curve presents the trade-off between the sensitivity (true-positive rate) and specificity (false-positive rate) of a classifier [66]. If a very stringent threshold is specified when determining a match to the motif, only the strongest true-positive sites will be identified, and the weaker matches will be missed. As the stringency of the match threshold is reduced, more true sites are identified at the expense of selecting more false-positive sites. An ROC curve allows us to examine how the false-positive and true-positive rates change as the threshold used to determine a match is altered. A useful score for integrating these two characteristics is the ROC-AUC score [67]. Intuitively, the closer the ROC-AUC is to 1.0, the better the motif. A score of 1.0 indicates that the motif is able to pick out all the true-positive sites with no false positives. If a motif is not able to do better than random, the ROC curve will be an approximately diagonal line, and the ROC-AUC score will be close to 0.5.

#### Clustering motifs eases analysis.

Analyzing large datasets with multiple motif discovery programs typically yields a large number of motifs. Even after filtering out spurious motifs that do not meet basic score-threshold requirements, there will often be many motifs left. These may correspond to subtle variants of a few distinct sequence signals present in the data. Thus, it is often desirable to cluster similar motifs together to reduce the total number of candidates to be validated. Clustering can be accomplished using any number of well-known algorithms [68], provided an appropriate similarity metric between motifs can be defined. The similarity calculation should take into account the fact that the motifs to be clustered may be of varying size, may represent overlapping but distinct regions of a larger motif, or may have reverse complementarity.

Harbison and colleagues used average squared distance between entries in the aligned PWMs, searching for the orientation and alignment that gave the minimum distance between motifs while enforcing a minimum overlap of seven nucleotides [39]. They then applied the k-medoids clustering algorithm [69] to the motifs. Kellis et al. clustered motifs using the fraction of common bits as a similarity metric. They applied hierarchical clustering to the motifs and combined clusters with a similarity exceeding 70% by computing a consensus sequence. A second, iterative, clustering step was then applied to combine motifs by co-occurrence in intergenic regions [49]. Similar procedures, using the Pearson correlation coefficient between motif PWMs as the similarity measure, have been applied [34,47]. Mahony and colleagues presented a method for clustering motifs using a self-organizing map [70]. Other sophisticated techniques have been developed specifically for clustering PWM motifs in the context of identifying co-regulated genes [71,72]. These methods could, in principle, be adapted for use as a postprocessing step in motif discovery.

#### Empirical significance testing and cross-validation reduce the risk of overfitting.

Although hypergeometric enrichment, ROC-AUC, and other scores can be very useful for comparing and ranking motifs, great care should be taken when trying to draw conclusions regarding the significance of the observed motifs. An arbitrarily complex motif model could produce motifs with ROC-AUC scores of 1.0, and huge statistical enrichment scores for any dataset. Even for relatively simple models, application of a motif discovery program to a particular sequence set may result in motifs that are severely overfit to the data. Spurious overrepresented patterns can be found in almost any dataset, and a motif obtained from a particular analysis with a very high hypergeometric enrichment score may not be any more statistically enriched than a motif learned by the same program from random data. To avoid these problems, we advocate two strategies: empirical significance testing and cross-validation.

Statistical significance can be assessed using randomized control calculations to calibrate the scores produced by a particular program. Controls are performed by running motif discovery on a large number of input sequence sets selected randomly from the genomic background [39,73] or generated according to some reasonable background model [63,70]. The motifs from each of these randomization runs are used to estimate an empirical score distribution. Using this distribution, a *p*-value can be assigned to a particular score by determining the empirical probability that the algorithm would produce a motif with the observed score (or better) from a random dataset equal in size to the input set. For each program, separate distributions should be generated for representative dataset sizes, as well as for motif models with different representational power (e.g., different lengths).

Overfitting can be addressed by performing motif discovery on a fraction of the data, and then using held-out test data to evaluate the motifs learned. This yields an unbiased estimate of how well the motif generalizes to unseen data. The variance of this estimate can be reduced using cross-validation. In cross-validation, the training and testing procedure is repeated for several training and test-set partitions. The measure of generalization performance can then be averaged across all trials. This approach is particularly applicable to discriminative motifs [74] used to build a classifier to distinguish bound from unbound sites. Two classification-based algorithms have recently demonstrated the utility of using cross-validation to protect against overfitting while learning motif models [12,27]. A review of cross-validation and other nonparametric techniques for estimating statistical error can be found in Efron et al [75].

#### Phylogenetic conservation information improves motif discovery performance.

Standard motif discovery programs perform well on bacteria and yeast sequence data, but perform relatively poorly on complex sequences from higher eukaryotes [58]. One way of augmenting sequence data to improve performance is by using orthologous sequences from related species. Transcription factor binding sites are important for ensuring proper control of gene expression, and therefore tend to be under selective pressure over evolutionary time. A significant fraction of evolutionarily conserved noncoding DNA has been shown to correspond to regions important for regulation [47,49,76–79]. One study found that 98% of known binding sites of skeletal muscle–specific transcription factors are confined to the 19% of human sequences most conserved in orthologous rodent sequences [78]. This tendency of transcription factor binding sites to be conserved across species has been exploited in the context of motif discovery by several different research groups.

One approach to leveraging conservation data is to identify blocks of sequence that are conserved across multiple species using phylogenetic footprinting [80,81]. Phylogenetic footprinting is a general technique for identifying conserved regions based on the evolutionary relationship among species. These conserved blocks can then be used as inputs to standard motif discovery tools and otherwise analyzed [71,72]. By culling only the conserved sequence from the input data, uninformative background DNA is eliminated, and an effective increase in signal to noise is achieved that facilitates the search for motifs [82].

Recently, several groups have developed motif discovery tools that integrate the ability to use conservation information directly into the motif search. One approach generated a catalog of motifs with potential regulatory importance by determining, on a genome-wide scale, which consensus sequences are highly conserved across species. Highly conserved motifs were validated by determining their overrepresentation among groups of co-regulated genes [47,49]. Many programs use an explicit, probabilistic model of evolution to relate orthologous sequences, and search for motifs using EM or a random sampling approach [73,83–86]. Other programs, while not explicitly modeling the evolutionary relationship between orthologous sequences, bias the motif search to highly conserved regions [87]. An alignment-based approach has been reported by Wang and Stormo that first generates profiles by aligning orthologous regions, and then merges similar profiles from nonorthologous regions to yield motifs [88]. The performance of state-of-the-art conservation-based methods is generally superior to standard motif discovery tools. In two recent re-analyses of the ChIP data from Harbison et al. [39], conservation-based tools demonstrated markedly better performance than the suite of six motif discovery tools used in the earlier study (some of which made simple use of conservation information) [73,86]. When alignments of orthologous sequences are available, using this data in concert with a conservation-based program will often improve performance, especially for highly degenerate or low-information-content motifs.

**Figure 3:**
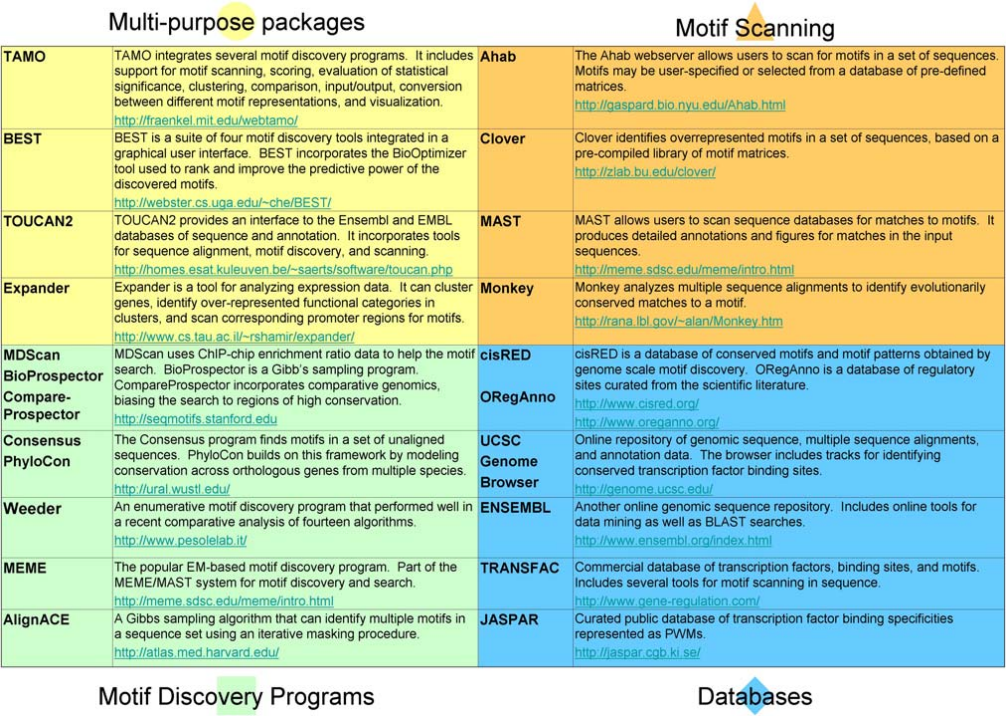
Resources

Phylogenetic conservation information may also be used to good effect when scanning sequences for putative transcription factor binding sites. While a good match to the motif is a very poor predictor of whether a site will be bound, matching sites that are also conserved in orthologous sequences are more likely to be functional. Very straightforward conservation thresholds on the number of matching orthologous sequences are easily applied, and have been used in generating maps of regulatory sites in yeast [39,73,89]. More sophisticated incorporation of the phylogenetic relationship among the aligned species has been used in concert with orthologous sequence data to search for putative regulatory sites [62]. When orthologous sequences in several species can be obtained, one can expect better motif discovery performance and more sensitive and specific identification of functional binding sites when scanning sequences. The use of these data is highly recommended. Some databases containing multiple sequence alignments, whole-genome sequences, and tools for performing cross-species motif analyses are listed in Figure 3.

## Interpreting the Biological Role of Motifs

Once an interesting set of motifs has been identified by motif discovery, the next logical step is to interpret the biological role of these sequence features. It may be possible to associate motifs with specific observable effects like up-regulation or down-regulation of gene expression in certain experimental conditions. Further biological insight into regulatory networks can be obtained by associating specific transcription factors with the motifs to which they bind. Standard motif discovery tools do not directly address these issues of interpretation. However, more recently, techniques have been developed that explore these questions.

### Motifs can be linked to their effect on gene expression using regression.

Regression-based techniques evaluate motifs by using them as features that predict the level of an interesting observable variable. These approaches may be particularly valuable when searching for regulatory motifs associated with expression changes in an experimental condition of interest. Bussemaker et al. presented the “regulatory element detection using correlation with expression” (REDUCE) method that enumerates the short DNA sequences present upstream of a set of genes, and then uses multivariate linear regression to associate gene expression level with the presence of these motifs [35]. Keles, van der Laan, and Eisen used motif enumeration with cross-validated feature selection to identify motifs that predict expression changes in a linear model [37]. Conlon and colleagues presented a similar method called “motif regressor” [36]. Their method extracts motifs using the MDScan program [40], filters out insignificant motifs, and then performs stepwise selection to build a multivariate model that predicts gene expression and identifies motifs that act together in regulatory programs. Smith and colleagues have recently presented a method that integrates motif discovery with regression to identify motifs, or pairs of motifs, that predict enrichment ratios in a ChIP-chip experiment [38]. Their method uses a modified linear regression model to first identify candidate motifs that correlate with binding from an initial set generated by motif discovery. A second motif discovery step identifies motifs located in close proximity to those in the candidate set. A nonparametric regression method is then used to identify interacting pairs.

### Discovering regulatory modules.

Complex regulatory programs may be realized through the action of combinations of transcription factors. Combinatorial control has been shown to be important in many contexts, including regulation of the cell cycle in yeast [90], sea urchin development [91], and the interferon-beta enhanceosome [92]. The binding sites of factors involved in combinatorial control are often clustered into cis-regulatory modules [93]. These cis-regulatory modules may have a biologically important structure that constrains both the number and relative position of the constituent motifs [94]. It is therefore of great interest to learn not only the representations of individual sequence motifs but also the higher-order structure of the modules into which motifs are organized.

Several algorithms have been developed that have the ability to search for pairs of interacting motif signals [38,63,95]. Other approaches to regulatory module discovery have used statistical tests or learning algorithms to identify overrepresented combinations from a previously generated set of motifs determined computationally or culled from literature sources [29,33,90,96]. These types of analyses often incorporate expression data, allowing motif combinations to be associated with particular regulatory programs. More recently, investigators have designed algorithms that learn cis-regulatory modules and the parameters of their constituent motifs de novo [97–100]. The tendency of the motifs to be clustered to a particular region, as well as statistical correlations in their positions within the module, can be exploited to improve the sensitivity of motif discovery [97]. These algorithms seem particularly promising since they may offer both improved performance over conventional motif discovery algorithms, as well as insight into the mechanism of regulation directed by the module's constituent transcription factors.

### Structural information can associate motifs with transcription factors.

Information about transcription factor structure and sequence can improve motif discovery results and reveal connections between specific transcription factors and motifs. The structure of a DNA-binding protein is closely linked to the motifs it binds. Proteins that dimerize, for example, often bind bipartite motifs with a low-information-content linker region, and specialized algorithms have been developed to take advantage of this knowledge [101,102]. More generally, it is possible to group transcription factors into families based on their structure and sequence [103]. Proteins from the same family tend to bind similar sequences, and Sandelin and Wasserman introduced the idea of biasing motif discovery toward motifs typical of the protein's family [53]. Related approaches have been proposed by Xing and Karp, who presented a Bayesian model of structural family characteristics for motif discovery [104], and by Mahony et al., who incorporated family binding profiles into a motif discovery algorithm based on the self-organizing map [105]. Structural information has also been used in the recently presented motif hypothesis–testing algorithm, THEME [27]. This study tested a series of binding specificity hypotheses derived from family binding profiles. Using a principled cross-validated approach, the THEME algorithm assigns an appropriate relative weighting to the initial hypothesis and the sequence information, performs a constrained optimization of the hypothesis, and evaluates the optimized motifs by their ability to correctly classify bound and unbound sequences.

The hypothesis-testing approach of THEME holds great promise for simultaneously learning both the family of the protein and the motif bound by that protein when neither is known in advance. MacIsaac and colleagues demonstrated that by testing the entire set of family binding profiles (representing 36 unique families) on the sequences bound in a ChIP experiment, they recovered the expected motif and the correct family as the top prediction for ten of 14 factors. In 13 of 14 cases, the correct prediction was ranked in the top five.

Tan, McCue, and Stormo have addressed the problem of connecting particular transcription factors to entries in a catalog of conserved motifs [106]. By computing a score measuring the average similarity of motifs to members of various DNA-binding families, Tan and colleagues calculated a probability that a transcription factor, from a known family, was associated with the correct motif. Combining this information with both phylogenetic and spatial data, motifs could be associated with the correct transcription factors in Escherichia coli with an impressive 85% accuracy rate for the top three predictions.

## Resources

Many excellent resources are available for analyzing sequence data with motif discovery, postprocessing motifs, and obtaining sequence and motif data. Freely available packages exist that integrate multiple motif discovery tools, and can greatly facilitate motif discovery analyses. Many stand-alone motif discovery tools are available in downloadable and Web-enabled form. Tools for motif scanning are often available with prepackaged libraries of known motifs, but also allow scans with custom motifs learned by motif discovery. Figure 3 contains some of these resources [15,24,30,40,54,61–63,87,88,107–119].

## Conclusions

Motif discovery can provide important insight into the mechanism of regulatory programs. Sophisticated tools and rich new data sources allow for greater success than ever before in learning motifs and in identifying in vivo binding sites. Recently developed techniques can help learn the context-specific effects of sequence motifs on gene expression, and offer the possibility of accurately associating specific proteins with discovered motifs. These advances open up the potential for building rich and accurate mechanistic models of genetic regulation.

There is a dizzying array of options available for undertaking sequence-based computational investigations, and experts can have very different opinions about the best tool or approach for a particular application. However, in our opinion, by following a few reasonable and simple guidelines, investigators can greatly increase their chances of successfully mining sequence data for motifs. Analyzing data with multiple motif discovery tools leverages the strengths of different algorithms and can greatly improve results. Postprocessing may include clustering to combine similar motifs and picking a common and intuitive scoring metric, but should always include a principled method for determining statistical significance of the motifs that takes into consideration the possibility of overfitting. Phylogenetic conservation information is useful both in aiding motif discovery and also as additional information used in distinguishing functional binding sites from spurious sites when scanning sequences. We recommend that this information be used wherever possible. Moving forward, we believe that cross-validated hypothesis testing and regression-based approaches will prove to be particularly valuable, as they combine the data-mining capabilities of classic motif discovery programs with a framework that offers an intuitive interpretation of the motifs. 

## Abbreviations

A: adenine
C: cytosine
ChIP: chromatin immunoprecipitation
EM: expectation maximization
G: guanine
ROC: receiver operating characteristic
ROC-AUC: area under the receiver operating characteristic curve
T: thymine
